# Continuing breastfeeding for at least two years after birth in rural Vietnam: prevalence and psychosocial characteristics

**DOI:** 10.1186/s13006-021-00427-8

**Published:** 2021-10-12

**Authors:** Hemavarni Doma, Thach Duc Tran, Tuan Tran, Sarah Hanieh, Ha Tran, Trang Nguyen, Beverley-Ann Biggs, Jane Fisher

**Affiliations:** 1 Global and Women’s Health, School of Public Health and Preventive Medicine, Monash University, Melbourne, Victoria Australia; 2 Research and Training Centre for Community Development, Hanoi, Vietnam; 3 Department of Medicine, University of Melbourne, Parkville, Victoria Australia; 4 The Victorian Infectious Diseases Service, Royal Melbourne Hospital, Parkville, Victoria Australia

**Keywords:** Sustained breastfeeding, Maternal age, Sex of child, Rural, Vietnam

## Abstract

**Background:**

The World Health Organization recommends breastfeeding for at least two years (24 months or more) after birth. In Vietnam, 22% of women continue breastfeeding for at least two years. The aim of this study was to determine the sociodemographic and psychosocial characteristics of mother-baby dyads associated with breastfeeding for 24 months or more in a rural setting in Vietnam.

**Methods:**

A secondary analysis was conducted on existing data obtained from a prospective study in Ha Nam, Vietnam. Women were recruited when they were pregnant and were followed up until 36 months after giving birth. The data were collected between 2009 and 2011. The associations between sociodemographic and psychosocial characteristics and continued breastfeeding for 24 months or more were examined using a multivariable logistic regression model.

**Results:**

Overall, 363 women provided complete data which were included in the analyses. Among those, 20.9% breastfed for 24 months or more. Women who were 31 years old or older were more likely to breastfeed for 24 months or more than women who were 20 years old or younger (adjusted odds ratio, AOR, 9.54 [95% CI 2.25, 40.47]). Women who gave birth to girls were less likely to breastfeed for 24 or more months than women who had boys (AOR 0.44; 95% CI 0.25, 0.80).

**Conclusions:**

This study provides evidence that may be useful for policy-makers to help improve breastfeeding practices for all children in Vietnam by targeting policy towards younger women and women with girls to promote continued breastfeeding for at least 24 months.

**Supplementary Information:**

The online version contains supplementary material available at 10.1186/s13006-021-00427-8.

## Background

Breastfeeding is a cost-effective source of nutrition that is essential in reducing morbidity and mortality as it confers many health benefits to both mothers [1] and children [2]. The World Health Organization (WHO) recommends that infants are fed only breastmilk for the first 6 months of life after which breastfeeding should be continued up to or beyond 2 years of age alongside nutritious complementary foods [3]. Meeting these recommendations is critical in preventing more than 800,000 child deaths per year [2].

Globally, less than half of children are breastfed for two or more years [4]. The prevalence of breastfeeding has been shown to vary based on the gross domestic product (GDP) of the country with low- and middle-income countries (LMICs) having longer breastfeeding durations than high-income countries [2]. In most high-income countries, less than 20% of women continue to breastfeed for 1 year [2]. In contrast, more than 70% of women are reported to continue breastfeeding for two or more years in certain LMICs such as Bangladesh, India and Nepal [5].

Breastfeeding practices are determined by characteristics at the community, household and individual level [6]. At the community level, social and cultural attitudes towards breastfeeding affect the duration of continued breastfeeding [6]. Breastfeeding can be viewed negatively especially when it occurs in public spaces and workplaces [7]. Healthcare providers play an important role in the maintenance of breastfeeding as they can influence mothers to opt for breastmilk substitutes as opposed to breastmilk [8]. This may in part be explained by gaps in knowledge about the importance of breastfeeding amongst healthcare providers [8, 9]. At the household level, factors such as the attitudes of some fathers and other relatives are influential in maintaining breastfeeding where some may support breastfeeding for a longer duration [10, 11]. Household wealth has also been shown to influence continued breastfeeding at 2 years in LMICs such as India and Pakistan, with those more disadvantaged found to breastfeed for longer periods [5].

At an individual level, employment type and education level can be associated with the early cessation of breastfeeding [6]. The intensity and practicality of employment can contribute to the decision to stop breastfeeding [12]. Additionally, the sex of the child can affect breastfeeding duration [13]. In some countries such as India, mothers prefer to breastfeed their boys longer than girls [14]. This preference may be due to mothers in India wanting to try again for a son therefore weaning their daughters earlier [14]. The relationship a mother has with her child in terms of controlling and settling their child can also cause a mother to cease breastfeeding [15]. They may assume that they do not have enough milk which may lead to the introduction of breastmilk substitutes [15]. Evidence also suggests that psychosocial characteristics such as depression throughout pregnancy [16, 17] and prenatal exposure to intimate partner violence [18] can predict early cessation of breastfeeding.

In Vietnam, 22% of women continued breastfeeding for at least 2 years [19] where breastfeeding practices are influenced by cultural and traditional beliefs of close relatives. For example, a woman’s husband may encourage breastfeeding for longer [20, 21]. Characteristics that have been proposed to be associated with breastfeeding cessation include lower maternal education level, women returning to work early or working further away from home, and inadequate support from negative influences of the breastmilk substitute market [21]. However, the possible influence of both sociodemographic and psychosocial characteristics on breastfeeding duration among women in Vietnam is not well known and understood [22]. Vietnam is a rapidly changing country shifting from lower-middle income to upper-middle income with a rate of the Gross Domestic Product per capital growth is among the highest in the world [23]. Important factors such as women’s participation in the workforce which is gradually changing from farming to the industrial sector, may affect their breastfeeding habits [24]. This study aims to examine the sociodemographic and psychosocial characteristics that can contribute to duration of breastfeeding for 24 months or more after birth among women in the rural province of Ha Nam, Vietnam.

## Methods

### Study design and setting

This study is a secondary analysis of the data collected from a population-based prospective study conducted in Ha Nam, a province in northern Vietnam in the Red River Delta [25].

Ha Nam is a rural province in northern Vietnam, approximately 50 km south of Hanoi. It has a population of approximately 880,000 people. Ha Nam has a health centre that is responsible for primary healthcare and implementation of national public health programs. Almost all women give birth in a medical facility (e.g., commune health centres, district hospitals, provincial hospitals) and receive standard care including free antenatal checks. They also have access to National Growth Monitoring and Expanded Immunisation Programmes [19]. The GDP of Vietnam in 2019 was 2715 USD per capita [23].

### Sample and participants

The population-based prospective study from which data for this study was extracted followed a cohort of women from early pregnancy to 3 years after birth and examined the effects of psychosocial and sociodemographic characteristics on early childhood health and development.

Participants of the original study were recruited through a two-stage sampling process. An independent statistician randomly selected 50 communes from a list of 116 communes in Ha Nam. A commune is the primary local government administrative unit in Vietnam. Each commune has a health centre and a population of 5000 to 10,000 residents. All pregnant women with a singleton foetus between 12- and 20-weeks’ gestation and residing in the selected communes between December 2009 to January 2010 were eligible to enrol in the study, and invited to participate [26].

Data were collected in six waves from 2009 to 2011. The first and second waves were conducted when the women were in early and late pregnancy, respectively. The third and fourth waves were conducted at eight weeks and six months postpartum, respectively. The fifth and sixth waves were conducted at 2- and 3-years postpartum, respectively.

Data were collected via study-specific, structured questions and psychometric measures. The psychometric measures were locally validated and standardised. Data collection was conducted by eight trained, blinded, and closely supervised health research workers from the Hanoi Research and training Centre for Community Development (RTCCD) in private rooms at commune health centres. Psychosocial data were collected through interview as opposed to self-report questionnaires as the latter were unfamiliar to most people. The design of this study has been reported elsewhere in greater detail [25].

A total of 498 women were recruited in the original study. Women who were known to have had multiple gestation pregnancy, multiple births, and a miscarriage or a stillbirth were excluded from this secondary data analysis. Women were also excluded from the analysis if their data on breastfeeding practices, or the psychosocial, demographic, economic and child characteristics were missing.

### Measures

#### Outcome

The duration of breastfeeding was assessed at the 2-year data collection wave through the questions ‘Are you currently breastfeeding?’ and ‘If not, how many months after birth did you stop breastfeeding?’. The responses of these questions were used to create the binary outcome variable: (1) mothers who were currently breastfeeding and/or have breastfed 24 months or more and (0) mothers who did not breastfeed or breastfed for < 24 months.

#### Psychosocial characteristics

Psychosocial characteristics were assessed at 24 months after childbirth.

Maternal mental health status was assessed using the Self Reporting Questionnaire (SRQ) which is an instrument developed by the WHO to screen for psychiatric disturbance especially among those in LMICs [27] and has been validated for use among mothers of young children in Vietnam [27]. The SRQ contains 20 yes/no ‘neurotic’ questions (e.g., do you feel unhappy? have you lost interest in things?) [27]. A total scale score that ranges from 0 to 20 (the number of ‘yes’ answers) indicates the severity of psychiatric disturbance [28].

Care given by mothers was measured by the Longitudinal Study of Australian Children Study’s Parenting Measure [29] that comprises three subscales: controlling the child’s behaviour (Control, 4 statements), expressing anger towards child (Anger, 5 statements), engaging with and consoling the child (Explanation, 9 statements). Responses to each statement posed to the women ranged from 0 ‘never/almost never’ to 4 ‘always/almost always’ with a maximum total score of 16, 20 and 36 for the control, anger and explanation subscales, respectively.

Intimate partner relationships were assessed using the Intimate Bond Measure (IBM) [30]. The IBM gauges two dimensions of the intimate partner relationship: Care and Control. Care assesses sensitivity, empathy, warmth, emotional responsiveness, capacity for companionship of the relationship. Control measures perceived criticism, coercion and dominance of the partner in the relationship. These two characteristics are negatively associated with each other whereby a high care would indicate low control. The IBM comprises 24 items (12 items for the care dimension and 12 for the control dimension). The scores on each subscale range from 0 to 36 with a score of 33 or more on the Care subscale indicating high or positive care and a score of 12 or more on the Control subscale indicating high or positive control. The comprehensibility and cultural relevance of the IBM have been validated in Vietnam for women who have just given birth or are pregnant [30].

#### Demographic and economic characteristics

Demographic characteristics were collected using study-specific questions at Wave 1. Maternal age was categorised into 25 years old or younger, 26 to 30 years old, and 31 years old or older. Level of education for mothers was categorised into completed primary school (Year 5) or lower; completed secondary school (Year 9); and completed high school (Year 12) or higher. For fathers’ level of education, we collapsed the lowest two categories into ‘completed secondary school or lower’ because the number of fathers who had completed primary school or lower was too small. Maternal occupation was categorised into farmer and non-farmer. Paternal occupation was categorised into farmer or not currently engaged in income-generating activity; factory worker, trader/self-employed, other or freelance; and government official/professional public or officer private services.

The hours mother spent away from child on the weekend and weekdays were also assessed through the questions ‘On average, how many hours are you away from the child on a weekday?’ and ‘On average, how many hours are you away from the child on a weekend day?’. The amounts were multiplied by five and two to obtain the total hours for five weekdays and one weekend, respectively.

Household wealth was assessed by the World Bank household wealth index method which calculated wealth from information collected on 17 household characteristics, services and durable assets using principal components analysis [31]. Each household characteristic was assigned a factor score generated through principal components analysis. The scores were then standardised with respect to a standard normal distribution with a mean household wealth value of zero and a standard deviation of one. Higher wealth index score indicated better household economic situation.

#### Child characteristics

The location of birth, birthweight, and child sex were collected from the birth certificate. Location of birth was categorised into provincial or district hospital; and commune health centre or at another location. Birthweight was left as a continuous variable.

### Data analysis

**Fig. 1:**
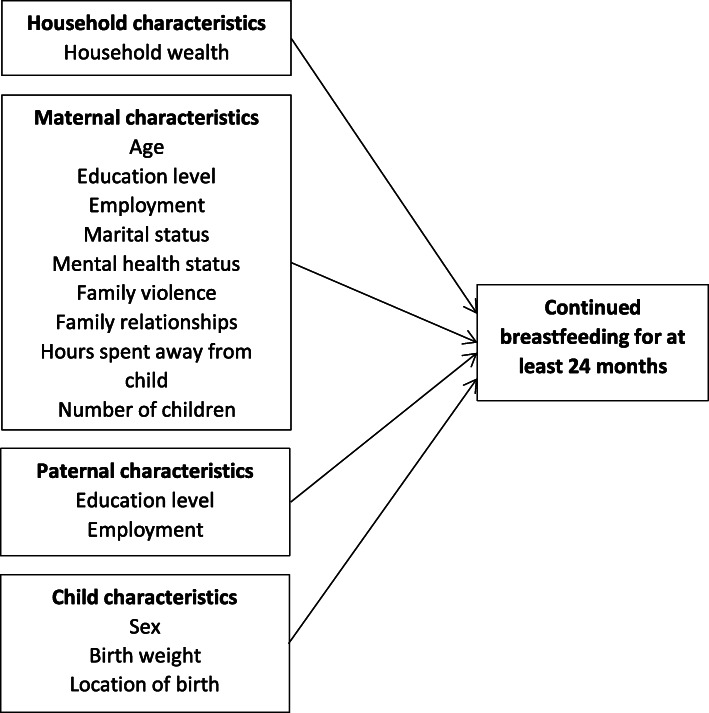
Conceptual framework demonstrating the sociodemographic and psychosocial characteristics assessed in this study

The analyses of this study were guided by a conceptual framework (Fig. 1) that was adapted from the framework on breastfeeding practices in South Asia [5], and our local knowledge [32]. Analyses were conducted in two stages. Stage 1 was the descriptive analyses of the sociodemographic and psychosocial characteristics of the sample. At Stage 2, the associations between these characteristics and breastfeeding at 24 months were examined simultaneously using a multivariable logistic regression model. Adjusted odds ratios and 95% CIs were reported. Analyses were performed using Statistical Package for the Social Sciences (SPSS) v26.0. Only participants with complete data were included in the analyses. Hosmer and Lemeshow tests were conducted to evaluate the goodness of fit for all the models. A *p* - value of the test < 0.05 was interpreted as a poor fit. Sensitivity analyses were performed using backward stepwise logistic regression analysis to verify the statistically significant associations found in the full model with smaller sets of variables.

## Results

### Sample

Of the 498 women who participated in the main prospective study, 135 (27.2%) women were not included for this study: 7 (1.4%) women had a miscarriage/or the baby was still born, 9 (1.8%) withdrew, and 119 (23.9%) were lost to follow-up or had missing data at 2 years postpartum. Data contributed by the remaining 363 (72.9%) women were included in the analyses.

The characteristics of the women are described in Table 1. About half were aged 25 years or younger. For two thirds of the women, the highest completed level of education was secondary school (Year 9). Slightly less than half of the mothers were farmers and 15% of fathers were farmers or not currently engaged in income-generating activity. Additionally, like the mothers, more than half the fathers had completed up to secondary school education.

**Table 1:** Sociodemographic and psychosocial characteristics of the participants (*N* = 363)

	Statistics
**Age, n (%)**	
25 years or younger	188 (51.8%)
26 to 30 years	106 (29.2%)
31 years or older	69 (19.0%)
**Mother’s education, n (%)**	
Completed primary school (Year 5) or lower	65 (17.9%)
Completed secondary school (Year 9)	195 (53.7%)
Completed high school (Year 12) or higher	103 (28.4%)
**Mother’s occupation, n (%)**	
Farmer	165 (45.5%)
Non-farmer	198 (54.5%)
**Father’s education, n (%)**	
Completed secondary school or lower	235 (64.7%)
Completed high school or higher	128 (35.3%)
**Father’s occupation, n (%)**	
Farmer or not currently engaged in income-generating activity	55 (15.1%)
Factory worker, trader/self-employed, freelance, or other manual work	278 (76.6%)
Government official/professional public, officer private services	30 (8.3%)
**Caregiving, mean (SD)**	
Control score	8.03 (2.11)
Anger score	5.68 (3.01)
Explanation score	24.95 (4.71)
**Number of children, n (%)**	
One child	264 (72.7%)
Two or more children	99 (27.3%)
**Hours spent away from child, mean (SD)**	
Weekday (hours/5 days)	27.74 (22.34)
Weekend (hours/2 days)	7.12 (8.88)
**Maternal mental health mean (SD)**	
Self-reporting Questionnaire score	3.36 (3.27)
**Birthweight (grams), mean (SD)**	3151.52(401.95)
**Child sex n (%)**	
Boy	200 (55%)
Girl	163 (45%)
**Location of birth, n (%)**	
Provincial or district hospital	210 (57.9%)
Commune health centre or at another location	153 (42.1%)
**IBM care, n (%)**	
Care score ≤ 32 (Low)	234 (64.5%)
Care score ≥ 33 (High)	129 (35.5%)
**IBM control, n (%)**	
Control score ≤ 11 (Low)	91 (25.1%)
Control score ≥ 12 (High)	272 (74.9%)

Around three quarters of mothers were primiparous. Approximately 60% of children were born in central or provincial hospitals and 42.1% of children were born in commune health centres (41.3%) or at another location (0.8%). The care given to the children was determined by three characteristics: control of child, anger towards child and explanation to child. For control over child, the average score was approximately 8 out of 16. For anger towards child, the average score was approximately 6 out of 20 and for explanation the average score was approximately 25 out of 36.

Additionally, the low average mental health score indicates that, overall, psychological well-being was high. With regards to intimate partner violence, less than 40% of the women had a high IBM Care score and 75% had a low IBM Control score.

### Breastfeeding for 24 months or more and associated characteristics

Of the 363 women, 76 (20.9% [95% CI 16.9, 25.5]) were breastfeeding their children at 24-months.

Women who were 31 years old or older were almost 10 times more likely to breastfeed for 24 months or more than women who were 20 years old or younger (Table 2). Women who had girls were more than 50% less likely to breastfeed for 24 months or more than women who boys.

**Table 2:** Multivariable logistic regression model of characteristics associated with breastfeeding for 24 months or more

	Breastfeeding for 24 months or more n (%)	Adjusted Odds Ratio	95% CI	
			Lower	Higher
**Age**				
25 years or younger	32 (17.0%)	1		
26 to 30 years	20 (18.9%)	1.26	0.63	2.51
31 years or older	24 (34.8%)	3.62	1.63	8.04
**Mother’s education**				
Completed primary school or lower	13 (20.0%)	1		
Completed secondary school	45 (23.1%)	1.52	0.68	3.42
Completed high school or higher	18 (17.5%)	1.48	0.52	4.24
**Mother’s occupation**				
Farmer	36 (21.8%)	1		
Non-farmer	40 (35.9%)	1.39	0.72	2.68
**Father’s education**				
Completed secondary school or lower	54 (42.6%)	1		
Completed high school or higher	22 (17.2%)	0.79	0.40	1.57
**Father’s occupation**				
Farmer or not currently engaged in income-generating activity	10 (18.2%)	1		
Factory worker, trader/self-employed, freelance, or other manual work	60 (21.6%)	2.21	0.90	5.43
Gov official/professional public, officer private services	6 (20.0%)	1.78	0.43	7.39
**Caregiving**				
Control score	Not applicable	0.93	0.81	1.07
Angry score	Not applicable	0.99	0.89	1.10
Explanation score	Not applicable	0.99	0.93	1.05
**Number of children**				
One child	60 (22.7%)	1		
Two or more children	16 (16.2%)	0.70	0.35	1.39
**Household wealth index**				
Lowest quartile (Poorest)	19 (21.1%)	1		
Second quartile	25 (26.6%)	1.31	0.61	2.81
Third and highest quartile (Richest)	32 (17.9%)	0.70	0.33	1.50
**Hours spent away from child**				
Weekday	Not applicable	1.04	1.00	1.09
Weekend	Not applicable	0.99	0.97	1.01
**Maternal mental health**				
Self-reporting Questionnaire score	Not applicable	1.34	0.58	3.06
**Birthweight (grams)**	Not applicable	1.00	1.00	1.00
**Child sex**				
Boy	53 (26.5%)	1		
Girl	23 (14.1%)	0.44	0.25	0.79
**Location of birth**				
Provincial or district hospital	36 (31.7%)	1		
Commune health centre or at another location	40 (26.1%)	1.88	1.05	3.39
**IBM care, n (%)**				
Care score ≤ 32	21 (23.1%)	1		
Care score ≥ 33	55 (20.2%)	0.83	0.44	1.57
**IBM control, n (%)**				
Control score ≤ 11	49 (20.9%)	1		
Control score ≥ 12	27 (20.9%)	1.08	0.60	1.95

Women who gave birth at a commune health centre or at another location were 88% more likely to breastfeed for 24 months or more than women who gave birth at a provincial or district hospital. Other characteristics were not statistically significantly associated with breastfeeding for 24 months or more.

The Hosmer and Lemeshow goodness of fit test yielded a *p* - value of 0.29 indicating the model adequately fits the data. From the sensitivity analysis, the significance of the variables age and child sex where unchanged in the stepwise logistic model (Additional file 1).

## Discussion

In this study, we examined the relationships between sociodemographic and psychosocial characteristics and continued breastfeeding for 24 months or more in a sample of women residing in rural Vietnam. Of the characteristics analysed, sex of child was found to be significantly associated with sustained breastfeeding. Mothers with daughters were less likely to be breastfeeding at 24 months postpartum than mothers with sons. Similar observations have been made in India where girls were breastfed for a shorter duration than boys [14]. In India, girls had a lower consumption of breastmilk by 21% than boys [33]. Furthermore, exclusive breastfeeding of boys was significantly higher at 70.8% than 61.5% of girls [34].

A possible reason for this observation could be related to the preference of sons over daughters similar to that observed in India and Pakistan. In India, a reason for breastfeeding girls significantly less than boys was attributed to mothers increasing their chance of having another child with the hopes of the child being a boy [14]. In Pakistan, the difference in breastfeeding duration between boys and girls is twice as high as in India which may be due to a stronger preference in sons than daughters [35]. This is consistent with Arnold [36] who states that the preference for sons is ten times greater than daughters in Pakistan but five times greater in India [36]. In Vietnam, the preference for sons is engrained in tradition. Particularly in North Vietnam, sons hold a central position in the family as they continue the patrilineal family line [37]. As a result, the process of family building is often planned around the need for sons. The birth of a son may then legitimise a woman’s position in her in-law’s family and the community [37]. Therefore, it may be the case that if a mother has a daughter, she may feel pressured to have a son. She may wean her daughter earlier in order to try to conceive a son. Mothers may also earn a higher status in the family if they have a son. Therefore, they may be able to allocate more time to taking care of their son as they may have less housework.

We also found that older women were more likely to continue breastfeeding for two or more years than younger women. In prior studies, higher maternal age has been associated with increased breastfeeding duration [38]. Similarly, younger mothers have been found to cease breastfeeding earlier than older mothers [13, 39–41]. A possible reason for such observations is that younger mothers may be more influenced by the breastmilk substitute industry [24]. Increase in intake of breastmilk substitutes has been associated with a decline in breastfeeding [42].

Breastmilk substitutes are still commonly used in Vietnam despite the implementation of policies such as the Decree on Trading In and Use of Nutritious Products for Infants (No. 21/2006/ND-CP) which prohibits advertising of complementary foods and breastmilk substitutes for children under six months and one year, respectively, to promote breastfeeding practices whilst regulating the baby formula marketing [43, 44]. Approximately half of newborn babies are fed formula within the first 3 days of life [45]. It is possible that older women are more discerning and may be more likely to exercise better judgement in breastfeeding practices including formula use. They may also have a stronger attachment to traditional practices and ways of infant feeding such as continued breastfeeding [46]. In contrast, younger mothers may have less insight and, therefore be more easily influenced by baby formula advertising and marketing.

Another reason young mothers may cease breastfeeding and opt for breastmilk substitutes could be a result of young mothers spending more time away from home. In rural Vietnam, younger women may have more opportunities to further their education compared to older women. This may result in higher paying occupations further away from home in cities like Hanoi [20]. Consequently, they may spend less time with their children which may influence them to opt for breastmilk substitutes [24]. Older mothers may have less access to highly skilled employment. Therefore, they may have more time to spend with their children and continue breastfeeding for a longer duration [24]. Older mothers may also require less support in breastfeeding than younger mothers. Lack of ongoing breastfeeding support especially in rural regions may negatively impact younger mothers’ decision continuing breastfeeding their children [47].

We also found that mothers who gave birth in commune health centres or at another location were more likely to continue breastfeeding for two or more years than mothers who gave birth in provincial or district hospitals evening after adjusting for socioeconomic status (other factors such as mother’s age, education and occupation; and father’s education and occupation); and sex of child. Women in Bangladesh were significantly more likely to initiate breastfeeding within the first hour of birth if they gave birth at home than if they gave birth in a facility [48]. In a hospital in Ho Chi Minh City, the prevalence of exclusive breastfeeding was lower than the national prevalence in Vietnam [49].

A reason for this observation could be the use of breastmilk substitutes in hospitals despite the introduction of policies aimed at reducing marketing of breastmilk substitutes [24]. Violations of breastfeeding policies aimed at reducing use of breastmilk substitutes are more prevalent in hospitals than commune health centres [50]. In a study of women in 11 provinces in Vietnam, 67% of families purchased breastmilk substitutes near the hospital or brought it from home compared to 39% of families who gave birth in a commune health centre [47]. Another reason for this observation could be health workers’ inadequate breastfeeding knowledge and skills especially regarding the WHO guidelines on infant and young child feeding practices [21, 51]. In this study, the education levels of both the women and their partners were not statistically significantly associated with continued breastfeeding when controlling for age and other characteristics. A previous study in Vietnam reported that woman’s education level was not associated with their uptake of information on breastfeeding practices and benefits [52]. This might suggest that the effectiveness of the current promotion strategies of breastfeeding in Vietnam is in the same level for the whole community. It could be both positive as effective for even the low educated women and negative as not effective for even high educated women.

Finally, we found the prevalence of continued breastfeeding in this rural province to be close to the national prevalence in Vietnam where approximately 22% of mothers continue to breastfeed for 24 months or more in Vietnam [25]. According to region, 24% of mothers breastfeed for 24 months or more in rural areas compared to 18% in urban areas [25]. The prevalence of continued breastfeeding for two or more years observed in Ha Nam is more closely reflective of the prevalence of continued breastfeeding for 24 months or more in rural areas of Vietnam [25].

### Strength and limitations

A strength of this research is the random selection of communes by an independent statistician which strengthened the representative adequacy of the study. Another strength is the use of locally validated standardised measures. This study also recruited almost all women who were eligible to be enrolled and included demographic characteristics on multiple levels (i.e., family and individual level) and psychosocial characteristics that were hypothesised to contribute to the continuation of breastfeeding for two or more years.

We acknowledge that that not all characteristics that could contribute to continued breastfeeding were accounted for as this was a secondary analysis of existing data. This study was conducted in a rural province. It is not representative of the whole population of Vietnam, especially the big cities. We also acknowledge that the rates of follow-up and missing data were relatively high in this study. As the missing data occurred in all major variables, listwise deletion (completed cases) was used in the study to handle the missing data. We believe that the missing data would not affect the results substantially because there was no indication that the missing were not at random.

## Conclusions

From this study, we have been able to demonstrate that younger women, women who give birth to girls, and women who give birth in provincial and district hospitals were less likely to breastfeed for 24 months or more which does not meet the WHO guidelines stating that breastfeeding should be continued for at least two years postpartum to achieve optimal growth and development for infants [3]. These pieces of evidence may assist policymakers in targeting policies towards younger women and women who have girls to increase breastfeeding duration. It may also assist policymakers in reviewing the healthcare that is provided to women in hospitals and emphasise the adherence to WHO guidelines on infant and young child feeding practices.

## Supplementary Information

**Additional file 1: Supplementary Table 1.** logistic stepwise (backwards elimination) regression model of characteristics excluding variables with *p* = 0.2. **Supplementary Table 2**: logistic stepwise (backwards elimination) regression model of characteristics excluding variables with *p* = 0.1.

## Data Availability

The datasets used and/or analysed for this study are available from the corresponding author on reasonable request.

## Abbreviations

CI: Confidence intervals
GDP: Gross domestic product
IBM: Intimate Bond Measure
LMICs: Low- and middle-income countries
RTCCD: Research and training Centre for Community Development
SD: Standard deviation
SRQ: Self Reporting Questionnaire
WHO: World Health Organization
