# Supplementary material for: Continuing breastfeeding for at least two years after birth in rural Vietnam: prevalence and psychosocial characteristics
Source: Int Breastfeed J. 2021 Oct 12;16:78. doi: 10.1186/s13006-021-00427-8 (PMC8507108; doi:10.1186/s13006-021-00427-8)
Supplement: Supplementary file 1 — Additional file 1: Supplementary Table 1. logistic stepwise (backwards elimination) regression model of characteristics excluding variables with p = 0.2. Supplementary Table 2: logistic stepwise (backwards elimination) regression model of characteristics excluding variables with p = 0.1. [file 13006_2021_427_MOESM1_ESM.pdf]

Supplementary Table 1: logistic stepwise (backwards elimination) regression model of characteristics excluding variables with p=0.2

	Adjusted Odds Ratio	95% CI	
		Lower	Higher
**Age**			
25 years or younger	1		
26 to 30 years	1.03	0.54	1.94
31 years or older	2.48	1.29	4.78
**Hours spent away from child**			
Weekday	0.99	0.97	1.01
Weekend	1.04	1.00	1.09
**Birth weight (grams)**	1.00	1.00	1.00
**Location of birth**			
Provincial or district hospital	1		
Commune health centre or at another location	1.79	1.05	3.05
**Child sex**			
Boy	1		
Girl	0.48	0.27	0.83

Variables entered on step one of stepwise logistic regression: age, mother's education, mother's occupation, father's education, father's occupation, caregiving, number of children, household wealth index, hours spent away from child, maternal mental health status, birth weight, child sex, location of birth, IBM care, and IBM control. The Hosmer and Lemeshow goodness of fit test p-value is 0.58.

Supplementary Table 2: logistic stepwise (backwards elimination) regression model of characteristics excluding variables with p=0.1

	Adjusted Odds Ratio	95% CI	
		Lower	Higher
**Age**			
25 years or younger	1		
26 to 30 years	1.05	0.56	1.99
31 years or older	2.63	1.37	5.03
**Location of birth**			
Provincial or district hospital	1		
Commune health centre or at another location	1.74	1.03	2.96
**Birth weight (grams)**	1.00	1.00	1.00
**Child sex**			
Boy	1		
Girl	0.48	0.28	0.83

Variables entered on step one of stepwise logistic regression: age, mother's education, mother's occupation, father's education, father's occupation, caregiving, number of children, household wealth index, hours spent away from child, maternal mental health status, birth weight, child sex, location of birth, IBM care, and IBM control, household wealth index, hours spent away from child, maternal mental health, location of birth. The Hosmer and Lemeshow goodness of fit test p-value is 0.44.
